# College Clinic

**Published:** 1859-03

**Authors:** 


*College Clinic.*—Service of Dr. JOHN. G. WESTMORELAND.

FEBRUARY 8.—The lecturer commenced the consideration of the subject of Diseases of the Respiratory Organs, and proposed first to call attention to the study of the physical diagnostic evidences, by which to determine not only the existence but particular character of those affections. In order to be able to determine the existence of disease he insisted upon a familiarity with the sounds discovered by percussion and auscultation in a healthy lung. The vesicular murmur, bronchial respiration, resonant sound on percussion, and natural expression communicated to the ear by the voice, was practiced by the class under his direction.

FEBRUARY 8.—The lecturer asked a continuation of the exercises in Auscultation and Percussion on the healthy subject, commenced on yesterday morning, calling attention also to the normal Auscultatory sounds of the heart.

A brief description given of abnormal respiratory and vocal sounds, and of those produced by percussion: 1stly. those produced in respiration, viz: Crepitation, Sibilant ronchus, Bronchial or Sonorous ronchus, and Cavernous ronchus. 2ndly. By the voice, as pectoriloquy, bronchophony and egophony. The particular states of the respiratory organs from which these unnatural sounds arise, also alluded to, and the importance of fixing in the mind these important facts previous to the exhibition of the cases in which the abnormal sounds will be practically studied.

FEBRUARY 9.—Judge, an old man affected with soft chancre, upon whom the operation for Phymosis was performed on the———— The wound looks well except near the frænum where the parts seem to be taking on the characteristic ulceration, but not to the extent which sometimes occurs after operations of the kind, during the progress of soft chancre of the phagadenic character. Symptoms resembling those of gonorrhea are now present, such as muco-purulent discharge and uneasiness in the urethra near the external orifice. This is probably the result of a specific ulcer, such as is found on the prepuce and body of the penis, within the canal. The attention of the class was called to the fact that often in cases of soft chancre this condition of the urethra is found, and the possibility of at least a similarity between the virus of gonorrhea and that of soft chancre.

FEBRUARY 10.—Two cases of pulmonary disease presented; Mrs. B., aged about forty-five years, the subject of disease of the lungs, probably from tuberculous deposit, for five years; and Mary, colored girl, previously examined before the class by Dr. Brown. As the object of this morning's clinic was to give practical demonstrations of the abnormal sounds on a subject in which they are distinct, the latter case alone was brought forward for this purpose, as Mrs. B. had so much improved as to render the sounds nearly natural. Each student was requested to examine the case as directed in a previous lecture and report for himself the character of the sounds and the nature of the lesion likely to produce them. Bronchophony, as the abnormal sound of the voice, want of full vesicular murmur and dullness on percussion were the evidences of disease in the left lung.—No very marked symptoms of derangement in the right were discovered—a slight sibilant rale, the only unnatural sound found.

